# Cracking the case: Seed traits and phylogeny predict time to germination in prairie restoration species

**DOI:** 10.1002/ece3.4083

**Published:** 2018-05-08

**Authors:** Rebecca S. Barak, Taran M. Lichtenberger, Alyssa Wellman‐Houde, Andrea T. Kramer, Daniel J. Larkin

**Affiliations:** ^1^ Plant Science and Conservation Chicago Botanic Garden Glencoe Illinois; ^2^ Program in Plant Biology and Conservation Northwestern University Evanston Illinois; ^3^ Program in Environmental Science University of Maryland, Baltimore County Baltimore Maryland; ^4^ Department of Environmental Science and Technology University of Maryland College Park Maryland; ^5^ Department of Fisheries, Wildlife, and Conservation Biology University of Minnesota St. Paul Minnesota

**Keywords:** community assembly, ecological restoration, germination, grassland, phylogeny, seed dormancy, seed traits

## Abstract

Traits are important for understanding how plant communities assemble and function, providing a common currency for studying ecological processes across species, locations, and habitat types. However, the majority of studies relating species traits to community assembly rely upon vegetative traits of mature plants. Seed traits, which are understudied relative to whole‐plant traits, are key to understanding assembly of plant communities. This is particularly true for restored communities, which are typically started de novo from seed, making seed germination a critical first step in community assembly and an early filter for plant establishment. We experimentally tested the effects of seed traits (mass, shape, and embryo to seed size ratio) and phylogeny on germination response in 32 species commonly used in prairie grassland restoration in the Midwestern USA, analyzing data using time‐to‐event (survival) analysis. As germination is also influenced by seed dormancy, and dormancy break treatments are commonly employed in restoration, we also tested the effects of two pretreatments (cold stratification and gibberellic acid application) on time to germination. Seed traits, phylogeny, and seed pretreatments all affected time to germination. Of all traits tested, variables related to seed shape (height and shape variance) best predicted germination response, with high‐variance (i.e., pointier and narrower) seeds germinating faster. Phylogenetic position (the location of species on the phylogenetic tree relative to other tested species) was also an important predictor of germination response, that is, closely related species showed similar patterns in time to germination. This was true despite the fact that all measured seed traits showed phylogenetic signal, therefore phylogeny provided residual information that was not already captured by measured seed traits. Seed traits, phylogenetic position, and germination pretreatments were important predictors of germination response for a suite of species commonly used in grassland restoration. Shape traits were especially important, while mass, often the only seed trait used in studies of community assembly, was not a strong predictor of germination timing. These findings illustrate the ecological importance of seed traits that are rarely incorporated into functional studies of plant communities. This information can also be used to advance restoration practice by guiding restoration planning and seed mix design.

## 1 INTRODUCTION

Functional traits are important predictors of how plant communities assemble and the ecosystem services they provide (Díaz & Cabido, 2001; Díaz et al., 2013; Laughlin, 2014; Roberts, Clark, & Wilson, 2010; Zirbel, Bassett, Grman, & Brudvig, 2017). The vast majority of studies that link functional traits to community assembly use vegetative plant traits of mature life stages—such as plant height and specific leaf area—to predict community outcomes. Regenerative traits that govern propagule production and dispersal, dormancy, germination, and establishment are vital to understanding assembly and persistence of plant communities, but are surprisingly understudied relative to traits of mature plants (Huang, Liu, Bradford, Huxman, & Venable, 2015; Jiménez‐Alfaro, Silveira, Fidelis, Poschlod, & Commander, 2016; Larson & Funk, 2016). This is a particularly important gap with respect to assembly of restored plant communities. Unlike most remnant plant communities, restorations are most often started from seed, making the transition from seed to germinant to established plant a highly influential process for restoration outcomes. Thus, seed traits may be as or more important than vegetative traits for understanding assembly of restored communities (Hoyle et al., 2015; Jiménez‐Alfaro et al., 2016; Larson & Funk, 2016). Improved understanding of seed and germination traits and their effects on plant germination, emergence, and establishment may help make restoration outcomes more predictable, a goal of restoration practice and research (Brudvig et al., 2017).

Seed germination is a critical life stage that drives assembly of restored plant communities (Larson, Sheley, Hardegree, Doescher, & James, 2015). Germination is irreversible, and therefore, early establishment is more sensitive to environmental variation than plant growth and survival in later life stages (Jiménez‐Alfaro et al., 2016). A seed that germinates at an inappropriate time may not survive to maturity, while dormant seeds face death by predation or disease (Clark & Wilson, 2003). Because of this, improved knowledge of germination responses is needed both to understand plant community assembly and to guide assembly via restoration planning, design, and practice.

Rapid germination, high overall germination, and the ability to germinate without cold stratification have been shown to impact establishment of species in restorations (Pywell et al., 2003). Furthermore, early‐germinating species can interfere with establishment, growth, or persistence of later‐germinating species, granting “priority” to early germinators. These priority effects can operate on very short timescales but have impacts that persist over many years (Young, Stuble, Balachowski, & Werner, 2017). Priority effects can not only favor early‐germinating native species over later‐germinating natives but also, and critically for restoration, impede establishment of invasive species (Grman & Suding, 2010; Young et al., 2017). Rapid germination and establishment of native species are desired outcomes for pre‐empting invasive species that are common in disturbed habitats and tend to have early germination phenology (Martin & Wilsey, 2012; McGlone, Sieg, & Kolb, 2011). To be sure, early germination is not the only important characteristic for establishment in restoration, and early germination can be detrimental if germinated seedlings are unlikely to establish and grow following germination. For example, in temperate systems, early germination of species that are not frost‐tolerant can be maladaptive (Leiblein‐Wild, Kaviani, & Tackenberg, 2014). Nonetheless, understanding factors that influence which seeds germinate and at what rates can help guide establishment of diverse restorations.

Seed mass is the most common and often the only seed trait used in functional ecology research due to its wide availability in trait databases and demonstrated importance for community dynamics (Jiménez‐Alfaro et al., 2016). Seed mass is related to plant functions such as seed dispersal, establishment, competition, frost tolerance, and plant growth rates (Kleyer et al., 2008; Leiblein‐Wild et al., 2014; Turnbull, Rees, & Crawley, 1999; Weiher et al., 1999; Westoby, Falster, Moles, Vesk, & Wright, 2002). Prior research indicates that seed mass can be positively or negatively predictive of germination (e.g., Kahmen & Poschlod, 2008; Norden et al., 2009) or not predictive at all (Shipley & Parent, 1991). Although seed mass is important for understanding community assembly, seed mass alone provides an insufficient basis for predicting differences in germination, establishment, and persistence (Larson & Funk, 2016).

External morphological traits like seed shape may be important for understanding germination and ultimately emergence and persistence. Seed shape has been linked to germination, with elongated seeds germinating more rapidly than rounded seeds (Bu et al., 2016; Grime, Mason, Curtis, Rodman, & Band, 1981). In some cases, seed shape has been a stronger predictor of germination than seed mass (Wang et al., 2016). In addition, seed shape is predictive of persistence in soil seed banks, with rounder seeds lasting longer than flat or pointed seeds (Thompson, Brand, & Hodgson, 1993).

Internal seed traits may also explain variation in germination, emergence, and persistence. For example, embryo‐to‐seed size (E:S) ratio, a measure relating the size of the embryo to that of the whole seed, is predictive of seed germination and establishment. Ecologically, E:S ratio was found to govern species’ establishment in multiple European habitats: low E:S genera tended to be found in moist areas while high E:S genera dominated dry habitats—likely because seeds with high E:S can germinate rapidly after imbibing water, an advantage in arid areas (Linkies, Graeber, Knight, & Leubner‐Metzger, 2010; Vandelook, Verdú, & Honnay, 2012).

Relationships between traits and germination are likely to exhibit phylogenetic signal, that is, closely related species are likely to have more similar trait values due to phylogenetic conservatism (Blomberg, Garland, & Ives, 2003). Such legacies of shared ancestry have been widely observed for seed mass (Moles et al., 2005; Norden et al., 2009). E:S is also a phylogenetically conserved trait; E:S ratios have generally increased over evolutionary time, with lower E:S ratios in basal angiosperms and higher ratios in younger clades (Forbis, Floyd, & de Queiroz, 2002). Because seed traits are likely to be phylogenetically conserved, simple regressions between traits and germination may be confounded by other factors that correlate with phylogeny. To isolate the effects of traits per se on germination, phylogenetic comparative methods can be used to account for the role of phylogeny on distribution of trait values (Pagel, 1999). Alternatively, rather than being statistically accounted for, phylogeny can be explicitly tested as a predictor variable. Phylogenetic measures can account for residual trait information that is phylogenetically correlated with but not captured by measured traits (Larkin et al., 2015; Pearse & Hipp, 2009). Phylogenetic position can also summarize key information about species in a way that integrates over many traits (Burns & Strauss, 2011; Cadotte, Cavender‐Bares, Tilman, & Oakley, 2009; Srivastava, Cadotte, Macdonald, Marushia, & Mirotchnick, 2012). Phylogenetic conservatism has been found to play a role in both seed traits and germination responses, and phylogeny can be used to understand variation in germination response that is not accounted for by measured seed traits alone (Bu et al., 2016; Hoyle et al., 2015; Seglias, Williams, Bilge, & Kramer, 2018; Wang, Baskin, Cui, & Du, 2009). Thus phylogenetic methods can both complement and strengthen inferences about the influence of traits.

Our goal was to test the degree to which seed traits and phylogeny were predictive of germination in a diverse set of plant species commonly used in ecological restoration of the North American tallgrass prairie. To do this, we conducted laboratory investigations tracking germination of individual measured seeds. We analyzed germination response using statistical time‐to‐event (survival) analysis with time to germination as the response variable and seed traits and phylogenetic position as predictor variables (McNair, Sunkara, & Frobish, 2012). In addition, because we suspected that seed dormancy would mediate the effects of seed traits and phylogeny on germination—and because seed pretreatments are a commonly used tool available to restoration practitioners to increase germination rates—we tested these relationships in seeds that were or were not subjected to treatments intended to break dormancy (cold stratification and gibberellic acid application). Finally, to disentangle the effects of traits and phylogeny on seed germination, we tested whether the traits we measured, and final germination percentages, showed significant phylogenetic signal. In sum, we tested the effects of seed traits, phylogenetic position, and germination pretreatment on time to germination of prairie plant species.

## 2 METHODS

### 2.1 Seed traits

**Figure 1:**
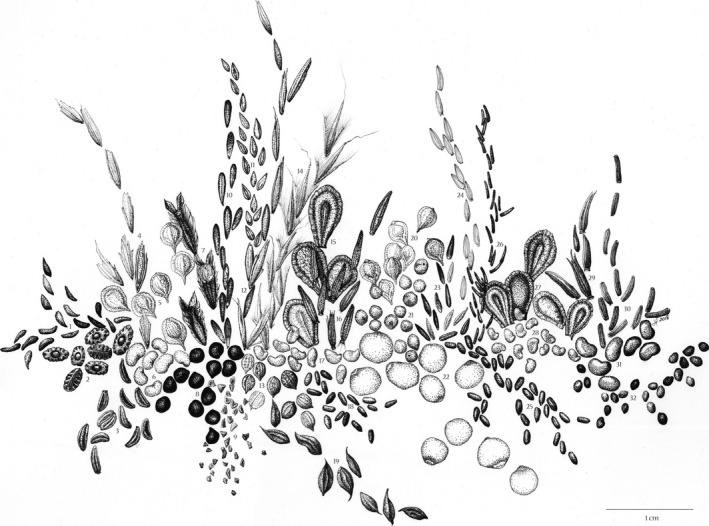
Drawing of the 32 prairie species in this study. Seeds are to scale. Artwork by Julia Ferguson. Species: 1. *Polemonium reptans*, 2. *Tradescantia ohiensis*, 3. *Zizia aptera*, 4. *Bromus kalmii*, 5. *Carex bicknellii*, 6. *Desmodium canadense*, 7. *Eryngium yuccifolium*, 8. *Sisyrinchium angustifolium*, 9. *Penstemon digitalis*, 10. *Symphyotrichum novae‐angliae*, 11. *Panicum virgatum*, 12. *Andropogon gerardii*, 13. *Euphorbia corollata,* 14. *Schizachyrium scoparium*, 15. *Asclepias syriaca*, 16. *Liatris scariosa*, 17. *Dalea candida*, 18. *Monarda bradburiana*, 19. *Thalictrum dasycarpum*, 20. *Carex brevior*, 21. *Sporobolus heterolepis*, 22. *Maianthemum racemosum*, 23. *Symphyotrichum laeve*, 24. *Solidago rigida*, 25. *Monarda fistulosa*, 26. *Rudbeckia hirta*, 27. *Asclepias verticillata*, 28. *Dalea purpurea*, 29. *Liatris spicata*, 30. *Vernonia gigantea*, 31. *Desmodium illinoense*, 32. *Anemone cylindrica*

We obtained seeds of 32 species (representing 26 genera and 14 families, Figure 1) that are commonly used in prairie restoration in the Midwest region, USA (Table 1) from Pizzo Native Plant Nursery (Leland, IL, USA); much of the sourced seed originated from Prairie Moon Nursery (Winona, MN, USA). Seeds were collected between 2014 and 2016, dried, and stored in a seed room at the nursery at low temperature and humidity. Additional information about the seeds, including dormancy status, cold stratification requirements and collection year and site can be found in Table S1. Upon receipt at Chicago Botanic Garden, dry seeds were refrigerated in the dark at 3°C until we initiated measurements and experiments.

**Table 1:** Plant species included in the study

Species	Family
*Andropogon gerardii*	Poaceae
*Anemone cylindrica*	Ranunculaceae
*Asclepias syriaca*	Apocynaceae
*Asclepias verticillata*	Apocynaceae
*Bromus kalmii*	Poaceae
*Carex bicknellii*	Cyperaceae
*Carex brevior*	Cyperaceae
*Dalea candida*	Fabaceae
*Dalea purpurea*	Fabaceae
*Desmodium canadense*	Fabaceae
*Desmodium illinoense*	Fabaceae
*Eryngium yuccifolium*	Apiaceae
*Euphorbia corollata*	Euphorbiaceae
*Liatris scariosa*	Asteraceae
*Liatris spicata*	Asteraceae
*Maianthemum racemosum*	Asparagaceae
*Monarda bradburiana*	Lamiaceae
*Monarda fistulosa*	Lamiaceae
*Panicum virgatum*	Poaceae
*Penstemon digitalis*	Plantaginaceae
*Polemonium reptans*	Polemoniaceae
*Rudbeckia hirta*	Asteraceae
*Schizachyrium scoparium*	Poaceae
*Sisyrinchium angustifolium*	Iridaceae
*Solidago rigida*	Asteraceae
*Sporobolus heterolepis*	Poaceae
*Symphyotrichum laeve*	Asteraceae
*Symphyotrichum novae‐angliae*	Asteraceae
*Thalictrum dasycarpum*	Ranunculaceae
*Tradescantia ohiensis*	Commelinaceae
*Vernonia gigantea*	Asteraceae
*Zizia aptera*	Apiaceae

We measured seed traits for each of 96 individual seeds per species, resulting in 3072 individually measured seeds. Measured traits comprised three broad categories: (1) seed mass, (2) seed shape, and (3) E:S ratio. We measured seed mass by weighing individual seeds using a precision balance. We characterized seed shape by measuring three dimensions (length, width, and height) using an ocular ruler on a dissecting microscope and by calculating variance as described in Kleyer et al. (2008). Lastly we measured E:S ratio using X‐ray analysis (Faxitron, Model MX‐W, Tucson, AZ, USA) to quickly and noninvasively measure the embryo relative to the whole seed, as has been used to measure seed embryos in crop species like cucumber (Gomes‐Junior, Chiquito, & Marcos‐Filho, 2013) and sunflower (da Rocha, Silva, & Cicero, 2014). We analyzed X‐ray images and calculated E:S ratio of each seed using imageJ software (Schneider, Rasband, & Eliceiri, 2012). We calculated E:S ratio in three ways: linear measures of embryo length and width relative to seed length and width, respectively, and embryo area relative to whole seed area. We used visual contrast to estimate embryo area and whole seed area, measuring the brightest part of each seed as the embryo. The three E:S measures are hereafter referred to as ES_length_, ES_width_, and ES_area_.

Prior to using seed traits as predictors in time‐to‐germination analyses, we tested for correlations among seed traits for each species using Pearson's product moment correlation coefficient. For pairs of traits that had Pearson's coefficient higher than 0.7, we selected one trait out of the pair and dropped the more redundant trait. We found two instances of trait correlations above 0.7 (Table S2), between mass and width (0.78), and between ES_length_ and ES_area_ (0.79). We retained mass as a predictor in the model selection process, and dropped width, because we had other measured shape variables (length, height, and shape variance), but only one for mass. We retained ES_area_ in the model, and dropped ES_length_, as area was a more inclusive E:S measure.

### 2.2 Germination

All 96 measured seeds of each species, as well as 48 unmeasured control seeds (to account for possible effects of handling and measurement on time to germination), were randomly assigned to three germination treatments: control, gibberellic acid, or cold stratification. Therefore, there were 32 measured and 12 unmeasured seeds of each species per treatment. Gibberellic acid (a plant growth hormone) and cold stratification are techniques to break seed dormancy (Baskin & Baskin, 2004; Johnson & Anderson, 1986) that are used in restoration practice (Rowe, 2010; Turner, Steadman, Vlahos, Koch, & Dixon, 2013).

We prepared 96‐well plates for germination by pouring a 2% agar solution into each well. Seeds were randomly placed in individual wells for germination. Separate 96‐well plates were used for each of the three treatments. Before being plated onto agar, seeds in the gibberellic acid treatment were placed into individual wells that did not contain agar and soaked in 500‐ppm gibberellic acid solution overnight (16–18 hr). Control seeds were soaked in water for the same duration. Seeds in the cold stratification treatment were placed in wells containing agar, covered with brown paper, placed in a cardboard box to keep out light and refrigerated (at 3°C) for 14 weeks to mimic overwintering conditions. We recorded locations within 96‐well plates to track individual seeds from pretreatment through germination, enabling us to obtain individual‐based germination data for seeds for which we also had complete trait data (i.e., measures of mass, length, height, shape variance, ES_width_ and ES_area_).

For germination assays, the 96‐well plates containing seeds were randomly positioned in an incubator set to a 12‐hr photoperiod with day/night temperatures of 20/10°C. Seeds were checked for germination (radical emergence of ≥1 mm, Meyer, Kitchen, & Carlson, 1995) three times each week for a total of 4 weeks. All germination tests and data collection took place between 23 June 2016 and 11 January 2017.

### 2.3 Phylogenetic tree

We constructed a phylogeny of the 32 species in this study by pruning a larger tree of 589 prairie plant species (Barak et al., 2017), which was modified from a published tree of 32,223 plant taxa (Zanne et al., 2014). The Zanne et al. (2014) tree was constructed based on GenBank sequences for seven gene regions (18S rDNA, 26S rDNA, ITS, matK, rbcL, atpB, and trnL‐F) using maximum likelihood for tree estimation. The Barak et al. (2017) tree was made by grafting species not present in the Zanne et al. tree and pruning nonfocal species using the weldTaxa and make.matandtree functions in the “Morton R project” (A. Hipp, Morton Arboretum, https://github.com/andrew-hipp/morton).

### 2.4 Data analysis

All analyses were performed using R version 3.3.1 (R Core Team 2016). The germination response variables were (1) a binary measure of whether or not a seed germinated and (2) the experimental day a seed germinated, with day 1 representing placement in the incubator and day 29 being the last day of the experiment. Predictor variables tested included seed traits, phylogenetic position, and germination pretreatment. Seed traits comprised six continuous measurements: mass, length, height, shape, ES_width_, and ES_area_.

Phylogenetic position was represented by quantitative, multivariate axes characterizing phylogenetic position for each species. To obtain these axes, we used a distance matrix of pairwise phylogenetic distances between each of the species in the experiment. We performed nonmetric multidimensional scaling (NMDS) ordination of the matrix using the isoMDS function in vegan (Oksanen et al., 2016) and extracted the position of each species along each of two axes. Germination pretreatment was a categorical factor with three levels: cold stratification, gibberellic acid, and a control group with no pretreatment.

We tested the effects of seed traits, phylogenetic position, and germination pretreatment on time to germination over the course of the experiment with time‐to‐event (survival) analysis using the survival package in R (Therneau & Grambsch, 2000). Survival analysis accounts for not only whether an event like germination occurs (a binary response) but also the amount of time it takes for the event to occur (a continuous response). We built survival models using a Cox proportional hazards model, which allows for both categorical and continuous predictors (McNair et al., 2012). Survival models were implemented using the coxph function in the survival package, with time to germination (in experiment days) as the response variable. Predictors were seed traits (6), phylogenetic position (2 NMDS axes) and germination pretreatment (categorical predictor with three factors). All continuous predictor variables were standardized prior to analysis (to mean = 0 and *SD* = 1) to produce standardized coefficients that could be readily compared among variables as indicators of effect sizes.

Candidate models comprising different combinations of predictor variables were constructed, and AIC‐based model selection was performed using the stepAIC function (MASS package, Venables & Ripley, 2002) on the time‐to‐event models. We performed stepwise model modification in both forward and backward directions and report top models (∆AIC ≤ 4). We performed model averaging on the top models using the modavg function in the package AICcmodavg (Mazerolle, 2016). We performed these analyses twice, once using all species in the experiment (*n *=* *32), and a second time excluding two species (*Maianthemum racemosum* and *Sisyrinchium angustifolium*) that had very low overall germination (< 5% germination in any treatment) to avoid undue influence of low‐germinating species on interpretation of results. We also used survival analysis to test for differences in germination response between measured seeds and unmeasured controls to evaluate whether measurements themselves introduced confounding error.

We tested for phylogenetic signal in the measured seed traits, that is, autocorrelation in species’ trait values that would be indicative of phylogenetic conservatism. We also tested for phylogenetic signal in final percent germination under each of the three pretreatments. Phylogenetic signal was evaluated with the *K* statistic using the phylosignal function in picante (Kembel et al., 2010). *K *=* *1 indicates the degree of phylogenetic signal in a trait that would be expected under a Brownian motion model of evolution, while *K *<* *1 and *K *>* *1 indicate lower and greater phylogenetic signal, respectively (Blomberg et al., 2003). Significance was assessed by comparing observed values of *K* to results from 1,000 permutations of tip‐shuffling randomizations.

## 3 RESULTS

Seed traits, phylogenetic position, and germination treatment were all retained in top‐ranking models for predicting germination (Table 2). The largest effect sizes of all predictors were germination pretreatments, the second phylogenetic axis, and shape variables including height and variance (Table 3). Shape variance was a positive predictor of time to germination, while height was a negative predictor. Taken together, these patterns are consistent with long, narrow seeds germinating more quickly, although length alone was not a strong predictor of time to germination. Unlike the shape variables mentioned, mass was not a strong predictor of time to germination. Measured and unmeasured seeds did not differ in time to germination, indicating that measurements were not confounding (*Z *=* *0.71, *p *=* *.48).

**Table 2:** Best models of time to germination ranked by Akaike information criterion (AIC) for 30 prairie species. *K* is the number of factors in the model, ∆AIC is the difference in AIC between each model and the model with the lowest AIC, *w* is the model weight and *Cw* is the cumulative model weight. Shown are all models with ∆AIC ≤ 4. Treat. = treatment, P1 and P2 =  multivariate phylogenetic axes 1 and 2, ES_width = _E:S ratio measured by width, ES_area = _E:S measured by area, L = length, W = width, H = height and VS = shape, measured as the variance between L, W and H

Model factors	*K*	AIC	∆AIC	*W*	*Cw*	*R* ^2^
Treat. + P1 + P2 + ES_width_ + ES_area_ + H + VS + Mass	9	19,319.68	0.00	0.43	0.43	0.15
Treat. + P1 + P2 + ES_width_ + ES_area_ + H + VS	8	19,320.51	0.83	0.28	0.71	0.15
Treat. + P1 + P2 + ES_width_ + ES_area_ + L + H + VS + Mass	10	19,321.53	1.85	0.17	0.88	0.15
Treat. + P1 + P2 + ES_width_ + ES_area_ + L + H + VS	9	19,322.24	2.56	0.12	1.00	0.15
~1 (Intercept‐only model)	0	19,772.37	452.69	0.00	1.00	

**Table 3:** Model‐averaged estimate, standard error, and 95% confidence interval (CRI) for all parameters in best fitting models (∆AIC ≤ 4) for 30 prairie species

Model term	Estimate	*SE*	95% CRI
Treatment—Cold stratified	0.85	0.07	0.71, 0.98
Treatment—Gibberellic acid	0.33	0.08	0.19, 0.48
Phylogenetic axis 1	0.13	0.03	0.07, 0.19
Phylogenetic axis 2	−0.41	0.04	−0.50, −0.33
Length	0.00	0.07	−0.13, 0.14
Height	−0.41	0.05	−0.50, −0.31
Shape variance	0.27	0.05	0.18, 0.37
ES_area_	0.09	0.03	0.03, 0.16
ES_width_	0.11	0.04	0.04, 0.18
Mass	−0.06	0.04	−0.14, 0.01

Final percent germination ranged from 0% to 94% depending on species and germination treatment (Table S3). Seeds of one species, *M. racemosum* (Asparagaceae), did not germinate under any germination treatments. Only a single seed of *S. angustifolium* (Iridaceae) germinated. In contrast, three species (*Dalea candida*,* Monarda bradburiana* and *Thalictrum dasycarpum*) reached 94% germination under gibberellic acid (*D. candida* and *M. bradburiana*) and cold stratification (*T. dasycarpum*) pretreatments. Of the 3,072 measured seeds, 15 did not contain embryos based on ES_area_ measures: four individuals of *Panicum virgatum*; two each of *Carex brevior*,* Solidago rigida,* and *Vernonia gigantea*; and one each of *Asclepias syriaca*,* Bromus kalmii*,* Eryngium yuccifolium*,* Liatris scariosa,* and *Polemonium reptans*.

As described above, we performed model selection using data from all species and with the low‐germinating species (*M. racemosum* and *S. angustifolium*) removed. Results based on 30 species (excluding low‐germinating species) are reported in the main text and those with all 32 species included are provided in Appendix S1. Interpretation of results and the effect sizes of model predictors were generally consistent between these two analyses. The main difference between the two models was that seed mass was a weak predictor of time to germination in the 30 species analysis, but was strongly negative in the averaged model based on all species; this is because *M. racemosum* had the heaviest seed of all species and never germinated.

**Figure 2:**
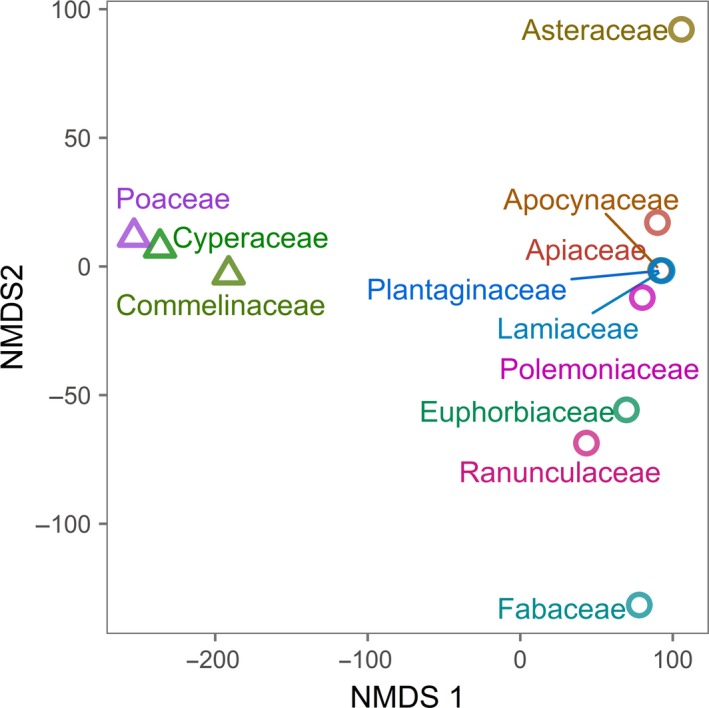
Nonmetric multidimensional scale (NMDS) ordination of phylogenetic distance matrix for 30 species that germinated in the study. Monocots are shown as squares and dicots as triangles. Only one point per family is shown, and points are color‐coded by family. NMDS includes two axes, stress = 15.71

**Figure 3:**
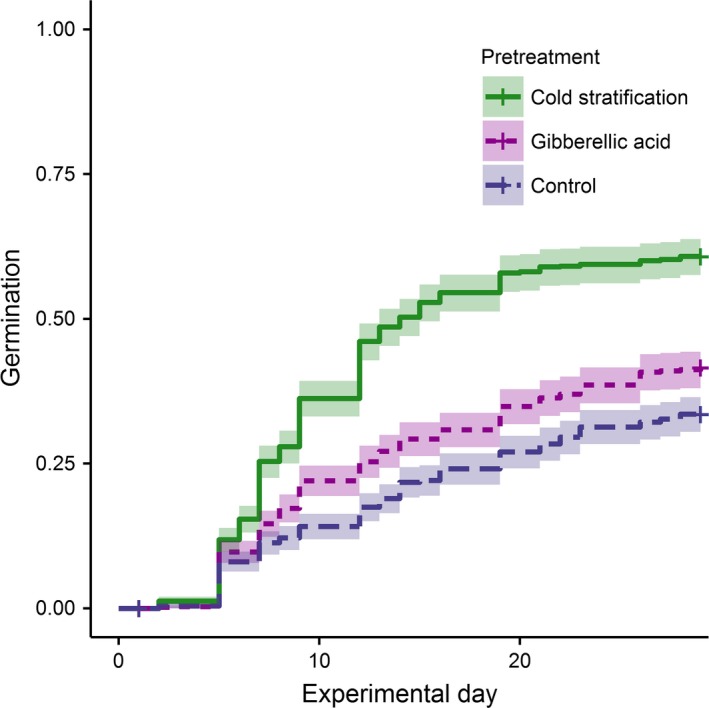
Time‐to‐germination curves under different seed pretreatments from Cox proportional hazards model

Phylogenetic NMDS ordination produced two axes describing phylogenetic position (stress = 15.71). NMDS axis 1 was strongly associated with the separation between monocots and dicots, and dicots (higher axis 1 values) tended to have higher germination. NMDS axis 2 moved across the phylogeny from Asteraceae to Fabaceae, with Fabaceae (lower axis 2 values) showing a stronger germination response (Figure 2). Both phylogenetic axes were predictors of time to germination, although axis 2 had a greater effect size in the averaged model. In general, germination responses were highest under cold stratification, which is necessary for dormancy break of many prairie species, followed by seeds treated with gibberellic acid, and finally control seeds (Figure 3).

**Figure 4:**
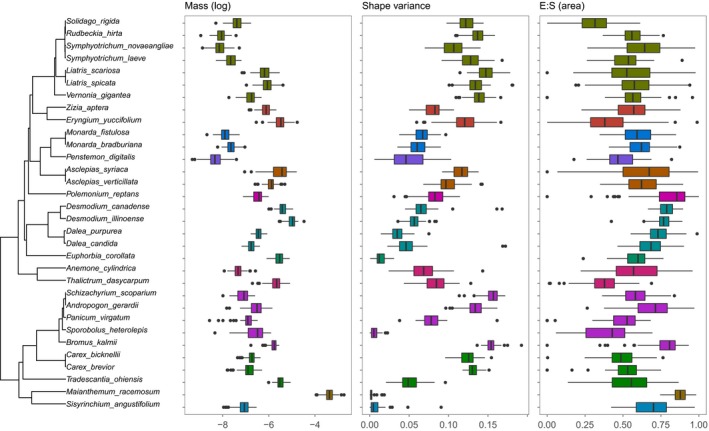
Phylogenetic tree of species used in the experiment and phylogenetic distribution of trait values representing seed size (mass), shape (variance), and embryo traits (ES_Area_). Color indicates plant family. The center of each boxplot is the median, while the boxes represent the first and third quartiles, and whiskers. All outliers greater than 1.5 times the interquartile distance (the length of the whiskers) are shown as individual points. All measured traits showed significant phylogenetic signal (see Table 4)

All seed traits showed low but significant phylogenetic signal, with *K* values ranging from 0.025 to 0.095 (Table 4, Figure 4). These values indicate higher phylogenetic signal than would be expected by chance but lower signal than expected under a Brownian motion model of evolution (Blomberg et al., 2003). Final percent germination showed significant phylogenetic signal under the control and gibberellic acid treatments, but not under the cold stratification treatment (Table 4).

**Table 4:** Phylogenetic signal of measured traits and final percent germination of 30 species under three germination treatments. *K* is the observed value of phylogenetic signal relative to a Brownian motion model of evolution. *P* is significance of phylogenetic signal based on a randomization test with 1,000 permutations

	*K*	*p*
Seed traits		
Length	0.071	.003
Height	0.064	.010
Shape variance	0.084	.003
ES_width_	0.033	.021
ES_area_	0.025	.060
Mass	0.095	.003
Percent germination		
Control	0.030	.038
Cold stratified	0.012	.392
Gibberellic acid	0.034	.025

## 4 DISCUSSION

Trait and phylogenetic measures were both necessary to explain differences in time to germination across 30 tallgrass prairie plant species. Despite phylogenetic effects being captured in part by measured seed traits that were phylogenetically conserved, phylogenetic position remained a significant predictor of time to germination. This indicates that phylogenetic position provided residual information not captured by measured traits alone—likely due to phylogenetic conservatism in biologically important but unmeasured traits, and/or phylogenetic measures being integrative across multiple traits and their interactions (Cadotte et al., 2009; Larkin et al., 2015; Pearse & Hipp, 2009; Srivastava et al., 2012).

We found that seed mass, the seed trait most commonly used in functional ecology and community assembly studies (Larson & Funk, 2016), was *not* one of the factors that best explained time to germination in our study species. Furthermore, we found seed mass to be a negative predictor of seed germination. While there is a theoretical expectation that seeds with higher mass should germinate faster, studies that encompass many species have shown the opposite—smaller seeded species often germinate more rapidly (e.g., Norden et al., 2009). Our findings underscore the importance of diversifying seed traits included in research on the assembly and functioning of plant communities (Larson & Funk, 2016). For example, shape‐based seed traits, which are simple and inexpensive to measure, had strong effects on time to germination and were retained in all top‐ranking models. Consistent with prior studies, we found higher germination rates in narrower seeds with higher shape variance (Bu et al., 2016; Grime et al., 1981). In addition, we found that embryo measurements were positive predictors of time to germination, that is, seeds with a larger embryo relative to the size of the whole seed germinated more rapidly. This consistent with the suggestion that seeds with a higher E:S ratio would germinate more rapidly after imbibing water (Linkies et al., 2010; Vandelook et al., 2012). While embryo measurements had lower explanatory power than shape‐based traits, we think there is potential for future study relating both seed shape and E:S variables to germination, emergence, and establishment of prairie species.

**Figure 5:**
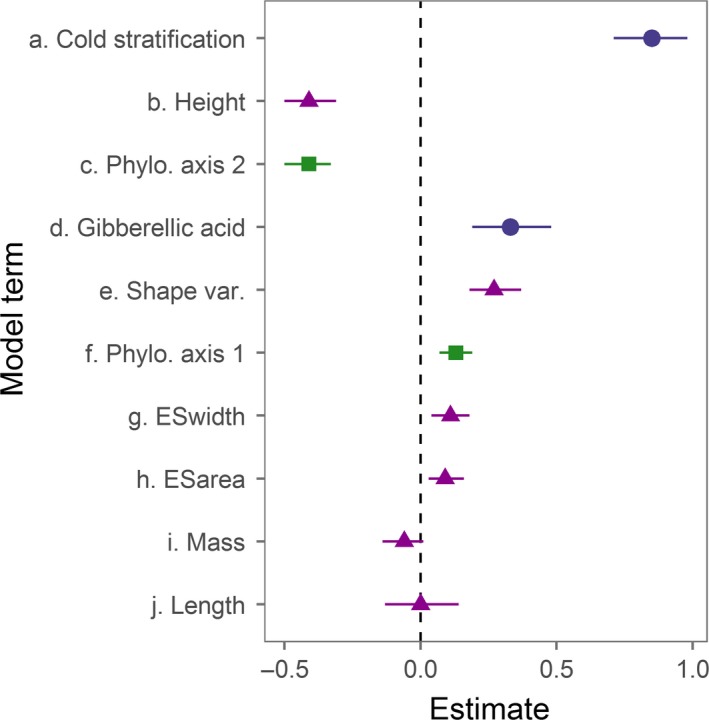
Estimates from averaged models (see Table 3), for germination pre‐treatment (blue circle), trait (purple triangle), and phylogenetic (green square) model terms. Error bars represent 95% confidence intervals (CRI)

Seed traits, dormancy patterns, and germination responses have ancient origins, and therefore, phylogenetic relationships remain an important part of understanding how they vary (Dayrell et al., 2016; Donohue, Rubio de Casas, Burghardt, Kovach, & Willis, 2010; Forbis et al., 2002; Linkies et al., 2010; Willis et al., 2014). Phylogenetic information was necessary for understanding differences in germination. This was true despite the fact that directly measured traits in our study themselves showed phylogenetic structure. That is, variance that might otherwise have been explained using phylogeny was already accounted for with trait measures. An example of the utility of including phylogenetic measures in our study was provided by the legume family (Fabaceae). In our experiment, species from the Fabaceae family germinated fairly rapidly under multiple pretreatments despite having rounder rather than longer and narrower seeds (Figures 2 and 5, Table S2). Including multivariate phylogenetic axes accounted for these and other clade effects that were unrelated to measures of seed mass, shape, and E:S ratio. Phylogenetic information also likely served as a proxy for unmeasured traits important for understanding germination responses (e.g., seed coat thickness or biochemical factors). Furthermore, phylogenetic information is integrative over evolutionary history and can be a stronger predictor of ecologically relevant information than traits alone (Hipp et al., 2015; Pearse & Hipp, 2009; Srivastava et al., 2012).

While we uncovered effects of seed traits, phylogeny, and pretreatment on germination response, there are opportunities to broaden this approach to include other considerations. For example, we did not vary germination temperatures, cold stratification lengths, or gibberellic acid concentrations. Varying these pretreatments would improve understanding of dormancy status and dormancy‐break requirements for the tested species. There are also opportunities for understanding how traits and phylogeny impact the range of possible germination responses (e.g., germination tolerance range), which may have implications for ecological restoration and predicting plant regeneration under climate change (Barak, Fant, Kramer, & Skogen, 2015; Jiménez‐Alfaro et al., 2016).

Furthermore, our experimental design accounted for individual differences between seeds, but we used only a single seed source for each species, all of which came from commercial nurseries. While each species used was collected only from one population, the collection locations differed across species, and we did not have precise location information for each species (Table S1). Thus, our study did not adequately account for population‐level effects on factors such as intraspecific variation in seed traits (e.g., Völler et al., 2012), timing of seed germination (Meyer et al., 1995), and dormancy (Seglias et al., 2018). While we did detect intraspecific variation in traits (Figure 4), explicitly addressing population‐level effects would provide additional insights into factors mediating seed germination and their implications for ecological restoration (Seglias et al., 2018; Violle, Castro, Richarte, & Navas, 2009; Völler et al., 2012). In addition, nursery growth (Gallagher & Wagenius, 2016) and storage conditions (including refrigeration) can have impacts on germination that we were unable to account for in our study. While our approach did accurately reflect how seed is commonly obtained, stored, and used in restoration, the results of our study should be interpreted with these caveats in mind.

This work has several implications for ecological restoration. First, we found that long and thin seeds germinated most rapidly. This information could be used in restoration design and management. For example, rapidly establishing native species could be preferentially seeded early on to establish cover of native species, conferring priority effects that could reduce invasion by undesired species (e.g., Young et al., 2017). Previous work demonstrates that seeds with these characteristics (i.e., high shape variance) do not form a persistent seed bank (Bekker et al., 1998; Thompson et al., 1993). Therefore, if species with elongated seeds do not germinate or establish early, they will likely need to be reseeded in later years. Repeated seeding has been shown to have positive biodiversity effects in restored prairies (Sluis, Bowles, & Jones, 2018). The effects of seed traits on germination, emergence, and establishment of prairie restoration species should be tested further to determine if our initial findings are robust to field conditions.

In addition, while we found that seed traits and phylogeny were important predictors of germination, pretreatment had a very strong effect on percent germination and time to germination in these species. While percent germination showed phylogenetic signal for nontreated and gibberellic acid‐treated seeds, seeds that had been cold stratified did not show phylogenetic signal in percent germination. Cold stratification increased percent germination in most species, so that their phylogenetic position became less relevant. It seems, then, that restoration managers are able to overcome, somewhat, the phylogenetic determinants of seed germination timing using cold stratification as a pretreatment methodology.

For prairie restoration, cold‐wet stratification is typically achieved in situ, by sowing seeds in the fall, so that they will emerge in the spring following a cold, wet winter. However, when fall planting is not possible, restoration practitioners have several options for increasing the likelihood of rapid germination and high proportions of germination overall. First, practitioners could sow seeds that are likely to germinate without cold stratification (e.g., in our study: *Andropogon gerardii*,* Anemone cylindrica*,* Bromus kalmii*,* Dalea candida*,* Dalea purpurea*, and *Rudbeckia hirta*, all of which had >75% germination without stratification). Second, practitioners could pretreat prior to seeding, using cold stratification indoors, or gibberellic acid, as we did in this study. In our study, gibberellic acid was less effective than cold stratification at accelerating germination, and it can have downstream effects on plant growth; nonetheless, it has the benefit that it requires much shorter durations than cold stratification. Taken together, preferentially planting species that germinate rapidly and to high percentages, and using pretreatments in the lab and field, may help grant priority to native species sown in restorations over invasive species (Young et al., 2017).

Larson et al. (2015) advocated for a trait‐based framework for understanding community assembly that can inform decision making for restoration. In particular, they suggested that traits relating to germination and emergence may drive restoration outcomes. However, a constraint to such a trait‐based approach is that only a fraction of traits that influence establishment are known and understood by researchers and managers (Larson et al., 2015). Here we demonstrate that seed traits—beyond seed mass—are predictors of germination response for a suite of species commonly seeded to restore prairie plant communities and that phylogeny helps explain germination response. Our findings support integrating additional traits and phylogenetic measures into germination studies as means to advance understanding of plant community assembly and to guide assembly through ecological restoration.

## CONFLICT OF INTEREST

None declared.

## AUTHORS’ CONTRIBUTIONS

R.S.B., T.L., A.W.H., A.T.K., and D.J.L. conceived the ideas and designed methodology; R.S.B. and T.L. collected the data; R.S.B. analyzed the data and led the writing of the manuscript. All authors contributed critically to the drafts and gave final approval for publication.

## DATA ACCESSIBILITY

All trait, phylogenetic, and germination data are available from the Dryad Digital Repository https://doi.org/10.5061/dryad.6301dq0.
