# Supplementary material for: Cracking the case: Seed traits and phylogeny predict time to germination in prairie restoration species
Source: Ecol Evol. 2018 May 8;8(11):5551–62. doi: 10.1002/ece3.4083 (PMC6010845; doi:10.1002/ece3.4083)
Supplement: Supplementary file 1 [file ECE3-8-5551-s001.docx]

Appendix S1: Effects of seed traits, phylogeny and germination pre-treatment on 32 prairie plant species (all species, including low-germinating species).

|  | Mass | Masslog | L | W | H | Variance | ES_Area | ES_length | ES_width |
| --- | --- | --- | --- | --- | --- | --- | --- | --- | --- |
| Mass | 1 | 0.726353 | 0.193333 | 0.867547 | 0.528349 | -0.35427 | 0.435541 | 0.279713 | 0.36761 |
| Masslog | 0.726353 | 1 | 0.526076 | 0.829244 | 0.667983 | -0.23095 | 0.375113 | 0.185532 | 0.185607 |
| L | 0.193333 | 0.526076 | 1 | 0.21141 | 0.578149 | 0.573819 | -0.00788 | -0.19429 | -0.08842 |
| W | 0.867547 | 0.829244 | 0.21141 | 1 | 0.581085 | -0.54059 | 0.261966 | 0.137535 | 0.195581 |
| H | 0.528349 | 0.667983 | 0.578149 | 0.581085 | 1 | -0.16819 | 0.196396 | 0.166523 | 0.153443 |
| Variance | -0.35427 | -0.23095 | 0.573819 | -0.54059 | -0.16819 | 1 | -0.22277 | -0.32126 | -0.23935 |
| ES_Area | 0.435541 | 0.375113 | -0.00788 | 0.261966 | 0.196396 | -0.22277 | 1 | 0.800277 | 0.71026 |
| ES_length | 0.279713 | 0.185532 | -0.19429 | 0.137535 | 0.166523 | -0.32126 | 0.800277 | 1 | 0.664249 |
| ES_width | 0.36761 | 0.185607 | -0.08842 | 0.195581 | 0.153443 | -0.23935 | 0.71026 | 0.664249 | 1 |

Table 1: Correlation table of traits with 32 species included.

Note: as ES_Area was highly correlated with both ES_length and ES_width, we used only ES_Area for the 32 species analyses.

Table 2. Best models of time-to-germination ranked by Akaike information criterion (AIC) for 32 prairie species. *K* is the number of factors in the model, ∆AIC is the difference in AIC between each model and the model with the lowest AIC, *w* is the model weight and *Cw* is the cumulative model weight. Shown are all models with ∆AIC ≤ 4. Treat. = treatment, P1, P2 = the multivariate phylogenetic axes 1 and 2, ES_area_ = E:S measured by area, L = length, W = width, H = height and VS = shape, measured as the variance between L, W and H.

| Model factors | *K* | AIC | ∆AIC | *W* | *Cw* | *R^2^* |
| --- | --- | --- | --- | --- | --- | --- |
| Treat. + P1 + P2 + ES_area_ + H + VS + Mass | 8 | 19416.58 | 0.00 | 0.71 | 0.71 | 0.18 |
| Treat. + P1 + P2 + ES_area_ + L + H + VS + Mass | 9 | 19418.39 | 1.81 | 0.29 | 1.00 | 0.18 |
| ~1 (Intercept-only model) | 0 | 20007.51 | 597.52 | 0.00 | 1.00 |  |
|  |  |  |  |  |  |  |

Table 3. Model-averaged estimate, standard error, and 95% confidence interval (CRI) for all parameters in best fitting models (∆AIC ≤ 6) for 30 prairie species. CS = cold stratified, GA = gibberellic acid treatment.

| Model term | Estimate | SE | 95% CRI |
| --- | --- | --- | --- |
| Treatment – Cold stratified | 0.84 | 0.07 | 0.70, 0.98 |
| Treatment – Gibberellic acid | 0.32 | 0.08 | 0.17, 0.47 |
| Phylogenetic axis 1 | 0.21 | 0.03 | 0.15, 0.27 |
| Phylogenetic axis 2* | 0.51 | 0.04 | 0.44, 0.59 |
| Length | 0.03 | 0.06 | -0.10, 0.15 |
| Height | -0.40 | 0.05 | -0.49, -0.30 |
| Variance (shape) | 0.43 | 0.05 | 0.34, 0.53 |
| ES_area_ | 0.13 | 0.03 | 0.07, 0.19 |
| Mass | -0.29 | 0.11 | -0.61, -0.17 |

*Note, phylogenetic axis 2 has a positive estimate in the 32 species analysis, but a negative estimate in the 30 species analysis. This is because the order of species along the phylogenetic axis flipped between the two analyses.

Table 4. Phylogenetic signal of measured traits and final percent germination under three germination treatments. *K* is the observed value of phylogenetic signal relative to a Brownian motion model of evolution. *P* is significance of phylogenetic signal based on a randomization test with 1,000 permutations.

|  | *K* | *P* |
| --- | --- | --- |
| *Seed traits* |  |  |
| Length | 0.072 | 0.002 |
| Height | 0.075 | 0.005 |
| Variance | 0.118 | 0.003 |
| ES_area_ | 0.030 | 0.042 |
| Mass | 0.115 | 0.001 |
| *Percent germination* |  |  |
| Control | 0.033 | 0.040 |
| Cold stratified | 0.016 | 0.227 |
| Gibberellic acid | 0.038 | 0.021 |


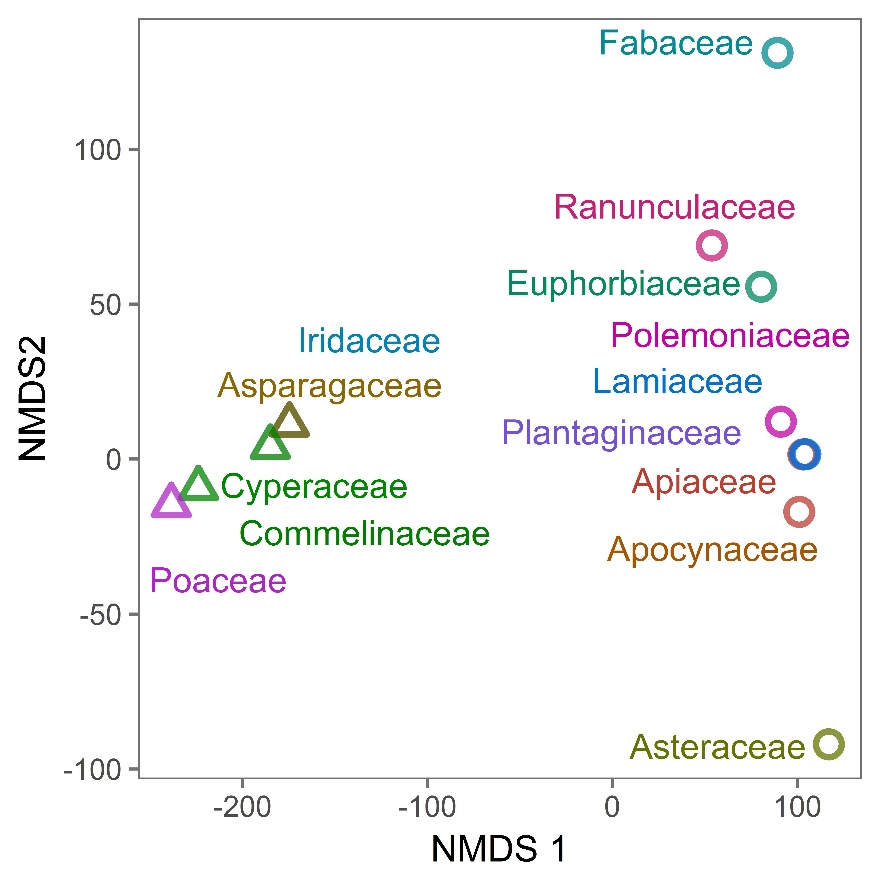


Figure 1. Non-metric multidimensional scale (NMDS) ordination of phylogenetic distance matrix for 30 species that germinated in the study. Monocots are shown as squares and dicots as triangles. Only one point per family is shown, and points are color-coded by family. NMDS includes 2 axes, stress = 16.99.
